# Supplementary material for: SNAP-25, but not SNAP-23, is essential for photoreceptor development, survival, and function in mice
Source: Commun Biol. 2024 Jan 5;7:34. doi: 10.1038/s42003-023-05760-8 (PMC10770054; doi:10.1038/s42003-023-05760-8)
Supplement: Supplementary file 2 — Supplementary Information [file 42003_2023_5760_MOESM2_ESM.pdf]

SUPPLEMENTARY INFORMATION

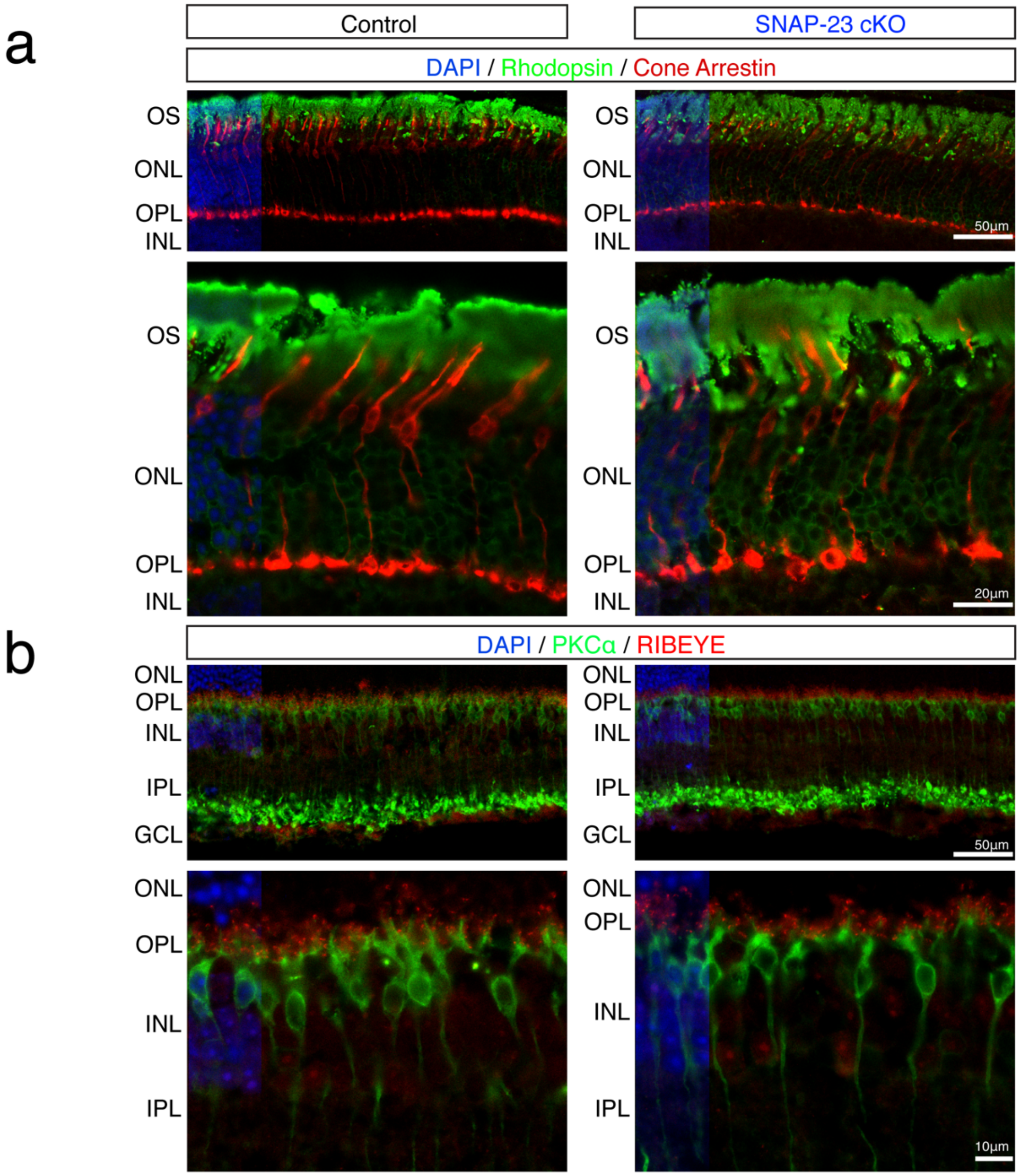

Supplementary figure 1. Removal of SNAP-23 does not alter retinal integrity.

(A) Immunostain of control and SNAP-23 cKO retinas for rods (green) and cones (red). Rods and cones are both present in SNAP-23 cKO retinas and stratify properly. Scale bar = 50 μm (left) and 20 μm (magnified, lower).

(B) Immunostain of control and SNAP-23 cKO retinas for rod bipolar cells (green) and synaptic ribbons (red). Synaptic ribbons are present in the presynaptic area of the synaptic area in both control and SNAP-23 cKO retinas. Synaptic ribbons form proper connections with post synaptic rod bipolar cells in both control and SNAP-23 cKO retinas. Scale bar = 50 µm (left) and 10 µm (magnified, lower).

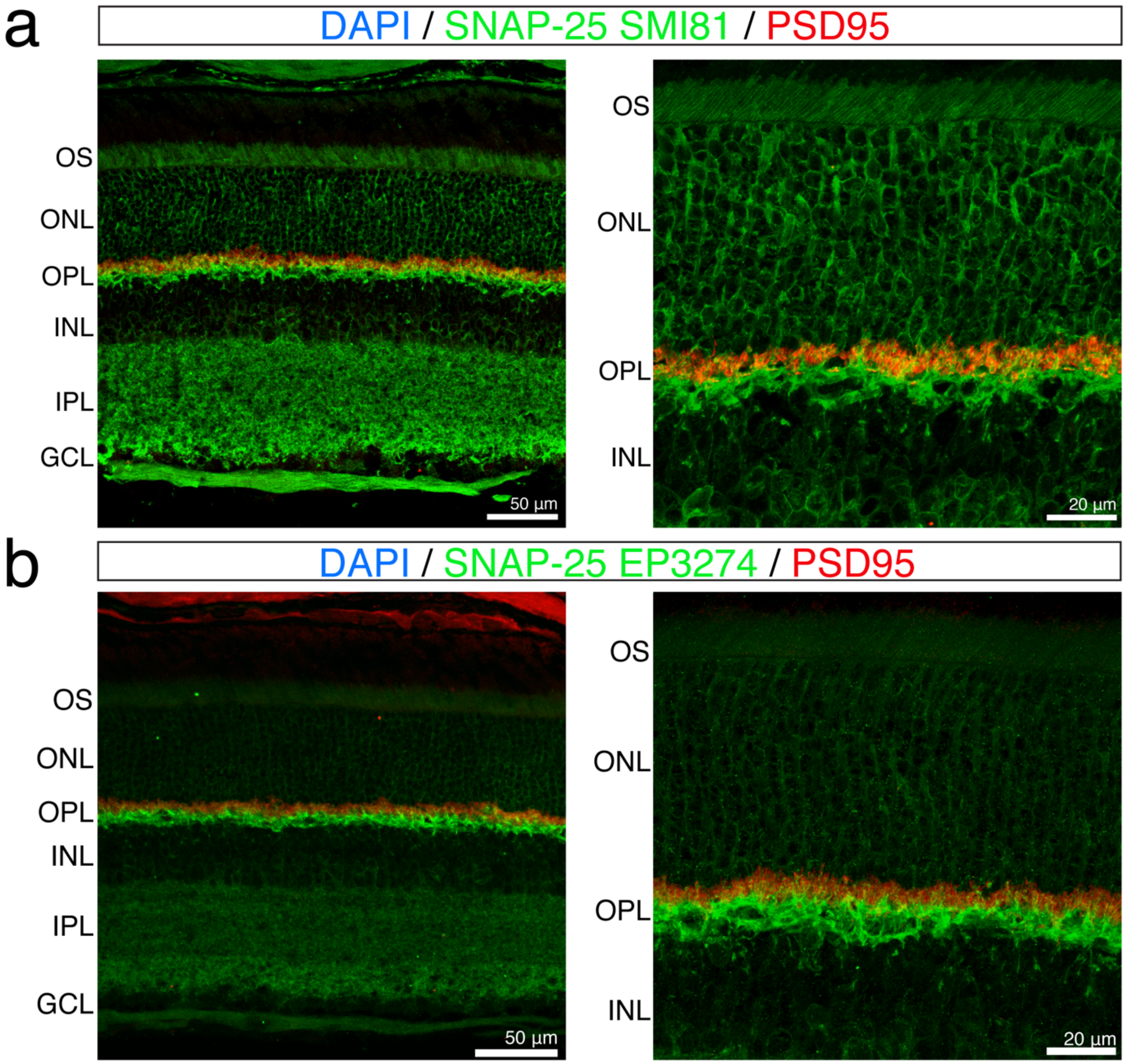

Supplementary figure 2. SNAP-25 protein can be detected by antibody staining.

(A) SNAP-25 SMI81 antibody staining (green) finds SNAP-25 throughout the retina, particularly in photoreceptor outer segments, surrounding photoreceptor nuclei, and in photoreceptor synaptic terminals. SNAP-25 colocalizes with presynaptic photoreceptor terminal marker PSD95 (red). SNAP-25 is also observed in the inner plexiform layer (IPL) and ganglion cell layer. Scale bar = 50 µm (left) and 20 µm (magnified, right).

(B) SNAP-25 EP3274 antibody staining (green) finds similar SNAP-25 presence in photoreceptor as with the staining observed in (A). Scale bar = 50 µm (left) and 20 µm (magnified, right).

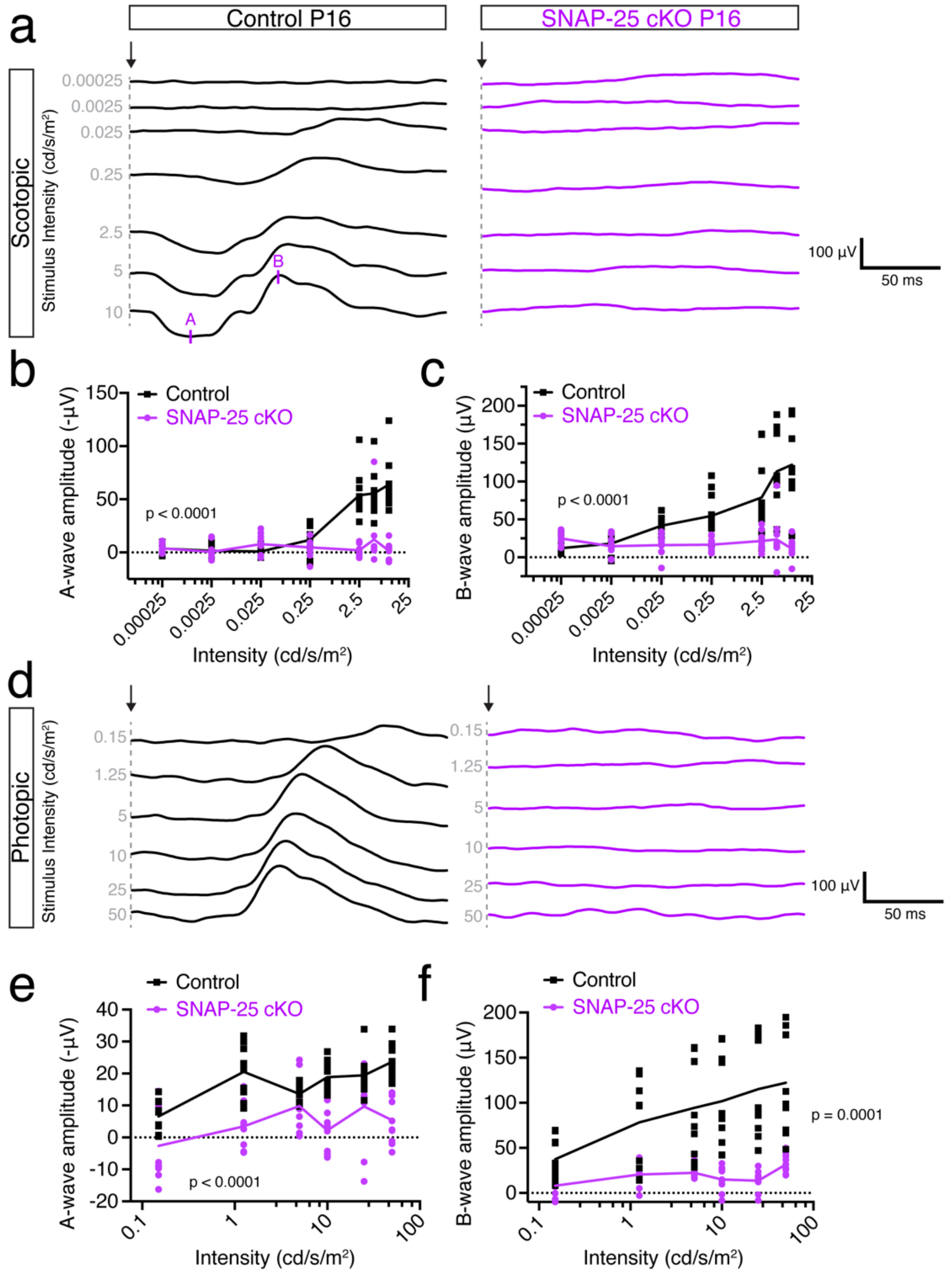

Supplementary figure 3. No visual function in SNAP-25 conditional knockout mice at postnatal day 16.

(A) Representative scotopic electroretinogram traces across 7 light intensities. Arrow indicate stimulation onset, A indicates a-wave, B indicates b-wave. SNAP-25 cKO mice exhibit no responses to light stimulation at any light intensity at postnatal day 16. Calibration bars represent 50 ms (horizontal), and 100 µV (vertical).

(B) Quantifications of scotopic a-wave amplitudes. (n = 5 mice for both control and SNAP-25 cKO for all quantifications; two-way ANOVA; $F_{(1, 18)} = 40.15$; $p < 0.0001$)

(C) Quantifications of scotopic b-wave amplitudes. Two-way ANOVA; $F_{(1, 18)} = 28.78$; $p < 0.0001$.

(D) Representative photopic electroretinograms across 6 light intensities. Arrow indicates stimulation onset. Calibration bars represent 50 ms (horizontal), and 100 μV (vertical).

(E) Quantification of photopic a-wave amplitudes; two-way ANOVA; $F_{(1, 18)} = 66.80$; $p < 0.0001$.

(F) Quantifications of photopic b-wave amplitude. Two-way ANOVA; $F_{(1, 18)} = 23.97$; $p = 0.0001$.

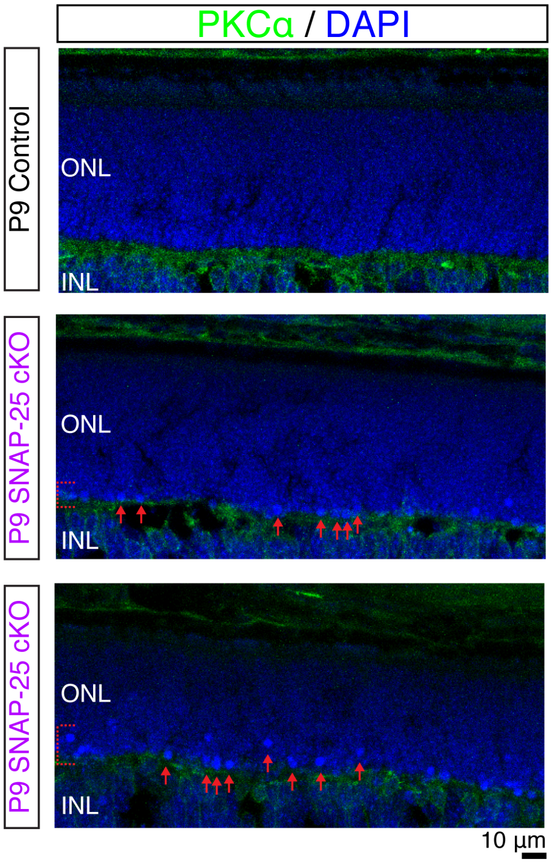

Supplementary Figure 4. Degeneration of DAPI binding in degenerating SNAP-25 cKO photoreceptors

DAPI (blue) and rod bipolar cell (PKCα, green) staining of retinal sections at P9. Altered nuclear permeability and changes in chromatin allowing increased amount of DAPI binding resulting in more intense DAPI signal can be observed in SNAP-25 cKO retina. Similar phenotype was observed across $n > 3$ mice per group. Scale bar = 10 μm.

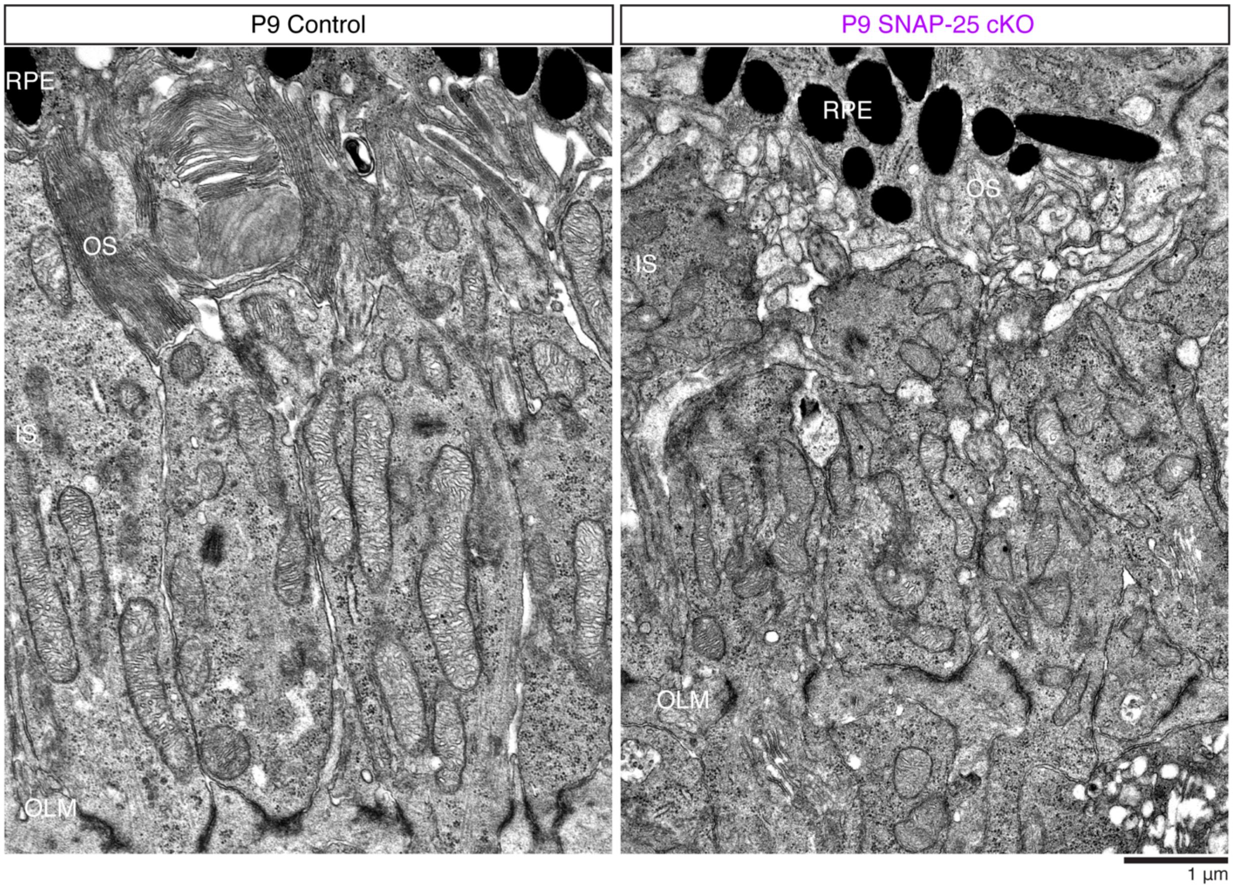

Supplementary figure 5. Disorganized outer segments in P9 SNAP-25 cKO mice.
Electron micrographs at 6000x magnification of control and SNAP-25 cKO P9 photoreceptor apical region. RPE (retinal pigment epithelium), OS (outer segment), IS (inner segment), OLM (outer limiting membrane). Scale bar = 1 μm.

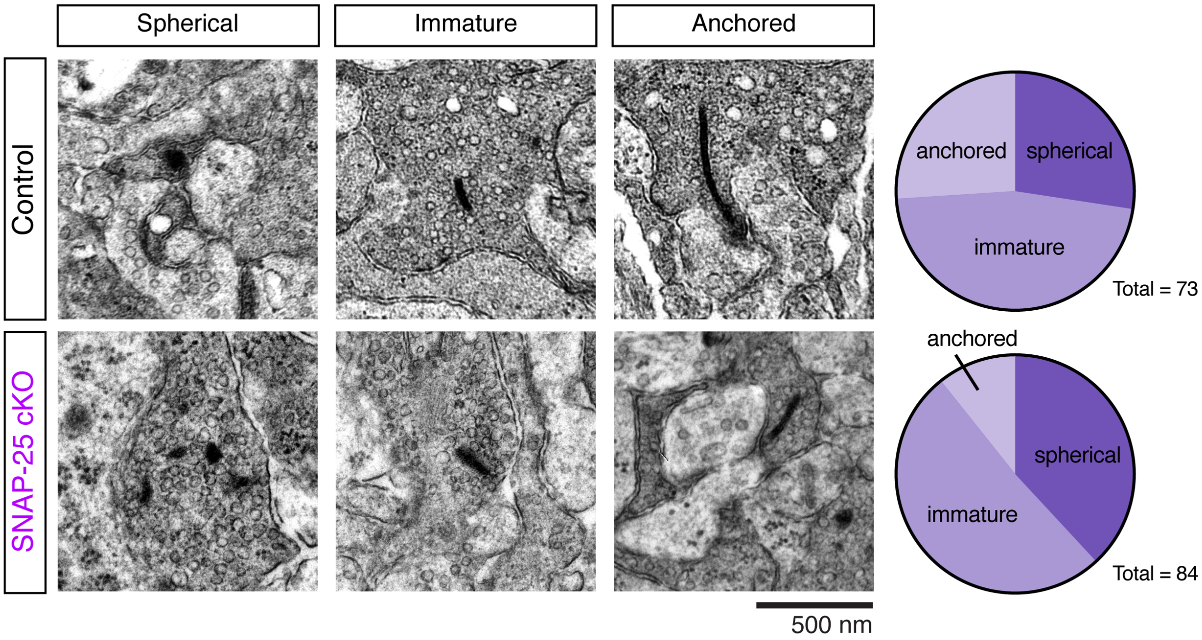

Supplementary figure 6. Changes in synaptic ribbon state in P9 SNAP-25 cKO retina. Synaptic ribbons were quantified in P9 control and SNAP-25 cKO mice according to their state: spherical, immature, and anchored. 70+ ribbons were scored as either spherical, immature ribbons (free floating), or anchored (with arciform density/opposed by at least two postsynaptic processes). More spherical ribbons were observed in SNAP-25 cKO retinas and there was a decrease in mature ribbons. Scale bar = 500 nm.

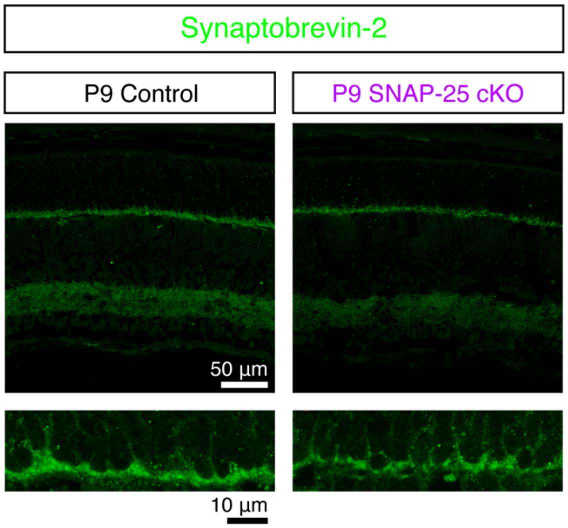

Supplementary figure 7. Normal expression of synaptobrevin-2 in control and SNAP25 cKO retinas at P9.

Immunostain of P9 control and SNAP-25 cKO retinas for synaptobrevin-2 (green). Synaptobrevin was observed presynaptically in photoreceptor terminals and in the inner plexiform layer. Scale bar = 10 µm.

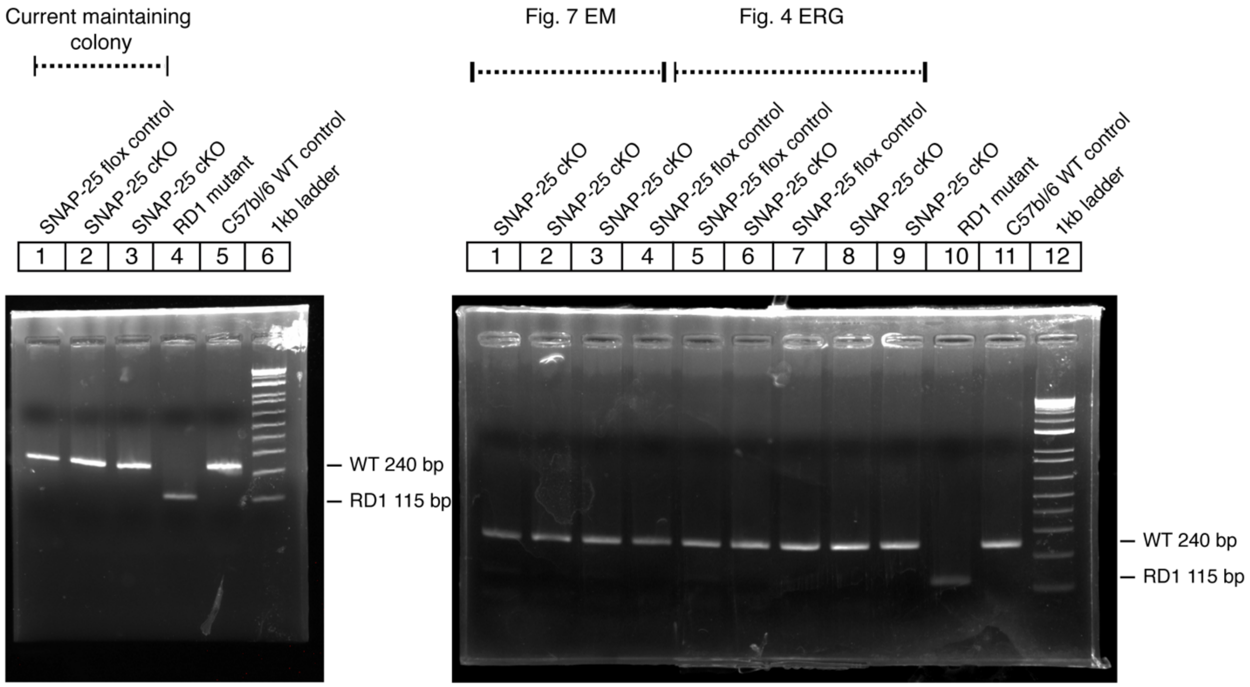

Supplementary figure 8. RD1 PCR confirming absence of RD1 mutant in our colony.
PCR of samples taken from our current maintaining mouse colony, as well as mice used in experiments detailed in this manuscript. Neither our current colony nor experimental mice show the 115 bp RD1 mutant amplicon that is present in RD1 positive controls.
