# Supplementary material for: SNAP-25, but not SNAP-23, is essential for photoreceptor development, survival, and function in mice
Source: Commun Biol. 2024 Jan 5;7:34. doi: 10.1038/s42003-023-05760-8 (PMC10770054; doi:10.1038/s42003-023-05760-8)
Supplement: Supplementary file 3 — Description of Supplementary Materials [file 42003_2023_5760_MOESM3_ESM.docx]

**Description of Additional Supplementary Files**

**File name:** Supplementary Data 1

**Description:** Source data underlying graphs and charts in the manuscript.
