# Supplementary material for: SNAP-25, but not SNAP-23, is essential for photoreceptor development, survival, and function in mice
Source: Commun Biol. 2024 Jan 5;7:34. doi: 10.1038/s42003-023-05760-8 (PMC10770054; doi:10.1038/s42003-023-05760-8)
Supplement: Supplementary file 5 — Reporting Summary [file 42003_2023_5760_MOESM5_ESM.pdf]

nature portfolio

Corresponding author(s): Shuzo Sugita

Last updated by author(s): Dec 19, 2023

Reporting Summary

Nature Portfolio wishes to improve the reproducibility of the work that we publish. This form provides structure for consistency and transparency in reporting. For further information on Nature Portfolio policies, see our Editorial Policies and the Editorial Policy Checklist.

Statistics

For all statistical analyses, confirm that the following items are present in the figure legend, table legend, main text, or Methods section.

n/a	Confirmed	
☐	☒	The exact sample size (*n*) for each experimental group/condition, given as a discrete number and unit of measurement
☐	☒	A statement on whether measurements were taken from distinct samples or whether the same sample was measured repeatedly
☐	☒	The statistical test(s) used AND whether they are one- or two-sided<br>*Only common tests should be described solely by name; describe more complex techniques in the Methods section.*
☐	☒	A description of all covariates tested
☐	☒	A description of any assumptions or corrections, such as tests of normality and adjustment for multiple comparisons
☐	☒	A full description of the statistical parameters including central tendency (e.g. means) or other basic estimates (e.g. regression coefficient) AND variation (e.g. standard deviation) or associated estimates of uncertainty (e.g. confidence intervals)
☐	☒	For null hypothesis testing, the test statistic (e.g. F, t, r) with confidence intervals, effect sizes, degrees of freedom and P value noted<br>*Give P values as exact values whenever suitable.*
☒	☐	For Bayesian analysis, information on the choice of priors and Markov chain Monte Carlo settings
☒	☐	For hierarchical and complex designs, identification of the appropriate level for tests and full reporting of outcomes
☒	☐	Estimates of effect sizes (e.g. Cohen's *d*, Pearson's *r*), indicating how they were calculated

Our web collection on statistics for biologists contains articles on many of the points above.

Software and code

Policy information about availability of computer code

Data collection	No software was used.
Data analysis	No code was used for data analysis.

For manuscripts utilizing custom algorithms or software that are central to the research but not yet described in published literature, software must be made available to editors and reviewers. We strongly encourage code deposition in a community repository (e.g. GitHub). See the Nature Portfolio guidelines for submitting code & software for further information.

Data

Policy information about availability of data

All manuscripts must include a data availability statement. This statement should provide the following information, where applicable:

- Accession codes, unique identifiers, or web links for publicly available datasets
- A description of any restrictions on data availability
- For clinical datasets or third party data, please ensure that the statement adheres to our policy

Data can be made available upon request to corresponding author.

Research involving human participants, their data, or biological material

Policy information about studies with human participants or human data. See also policy information about sex, gender (identity/presentation), and sexual orientation and race, ethnicity and racism.

Reporting on sex and gender	Both male and female mice were used for this study.
Reporting on race, ethnicity, or other socially relevant groupings	N/A
Population characteristics	N/A
Recruitment	N/A
Ethics oversight	N/A

Note that full information on the approval of the study protocol must also be provided in the manuscript.

Field-specific reporting

Please select the one below that is the best fit for your research. If you are not sure, read the appropriate sections before making your selection.

☒ Life sciences ☐ Behavioural & social sciences ☐ Ecological, evolutionary & environmental sciences

For a reference copy of the document with all sections, see nature.com/documents/nr-reporting-summary-flat.pdf

Life sciences study design

All studies must disclose on these points even when the disclosure is negative.

Sample size	For all quantified tests (OCT, ERG, OKT), minimum triplicates were used at every time point.
Data exclusions	No data were excluded from analysis.
Replication	Reproducibility was confirmed using triplicates.
Randomization	No randomization
Blinding	No blinding

Reporting for specific materials, systems and methods

We require information from authors about some types of materials, experimental systems and methods used in many studies. Here, indicate whether each material, system or method listed is relevant to your study. If you are not sure if a list item applies to your research, read the appropriate section before selecting a response.

Materials & experimental systems

n/a	Involved in the study	
☐	☒	Antibodies
☒	☐	Eukaryotic cell lines
☒	☐	Palaeontology and archaeology
☐	☒	Animals and other organisms
☒	☐	Clinical data
☒	☐	Dual use research of concern
☒	☐	Plants

Methods

n/a	Involved in the study	
☒	☐	ChIP-seq
☒	☐	Flow cytometry
☒	☐	MRI-based neuroimaging

Antibodies

Antibodies used	1. Rabbit monoclonal SNAP-23 Novus Biologicals Cat #. NBP2-67157<br>2. Mouse monoclonal SNAP-25 Covance SMI-81R<br>3. Rabbit monoclonal SNAP-25 Abcam EP3274<br>4. Rabbit PSD95 Abcam 18258

	5. Mouse PSD95 Abcam 2723<br>6. Mouse Calbindin D281K Sigma CB-955<br>7. Mouse PKCα Santa Cruz sc-8393<br>8. Guinea Pig RIBEYE Synaptic Systems 192 104<br>9. Mouse Rhodopsin B630 Novus Bio NBP2-25160<br>10. Rabbit Cone-arrestin Millipore 15282<br>11. Rabbit S-opsin Novus Bio NBP1-20194<br>12. Guinea Pig Synaptobrevin-2 Synaptic Systems 104 204
Validation	Verification citations<br>1) Huang M, et al. 2023. iScience. (IHC)<br>2) Hirano AA, et al. 2011. J. Comp. Neurol. 519:972. (WB, IHC, EM) PubMed<br>3) Knockout validated supplier website https://www.abcam.com/products/primary-antibodies/snap25-antibody-epr3275-ab109105.

Animals and other research organisms

Policy information about studies involving animals; ARRIVE guidelines recommended for reporting animal research, and Sex and Gender in Research

Laboratory animals	Mouse: SNAP23tm1Jpes (SNAP-23 flox)<br>Mouse: B6-Snap25tm3mcw (SNAP-25 flox)<br>Mouse: Tg(Crx-cre)764Gla (CRX-cre)<br>Mouse: SNAP23tm1Jpes ; Tg(Crx-cre)764Gla (SNAP-23 cKO)<br>Mouse: B6-Snap25tm3mcw; Tg(Crx-cre)764Gla (SNAP-25 cKO)
Wild animals	N/A
Reporting on sex	Mice of both sex were used for this study
Field-collected samples	N/A
Ethics oversight	All experiments detailed here were reviewed and approved by the animal care committee of the University Health Network in accordance with the Canadian Guidelines for Animal Care.

Note that full information on the approval of the study protocol must also be provided in the manuscript.
